# Supplementary material for: Functional Availability of ON-Bipolar Cells in the Degenerated Retina: Timing and Longevity of an Optogenetic Gene Therapy
Source: Int J Mol Sci. 2021 Oct 26;22(21):11515. doi: 10.3390/ijms222111515 (PMC8584043; doi:10.3390/ijms222111515)
Supplement: Supplementary file 1 [file ijms-22-11515-s001.zip › Figure S1.pdf]

Timeline of retinal degeneration in C3H/HeOu rd1 mice

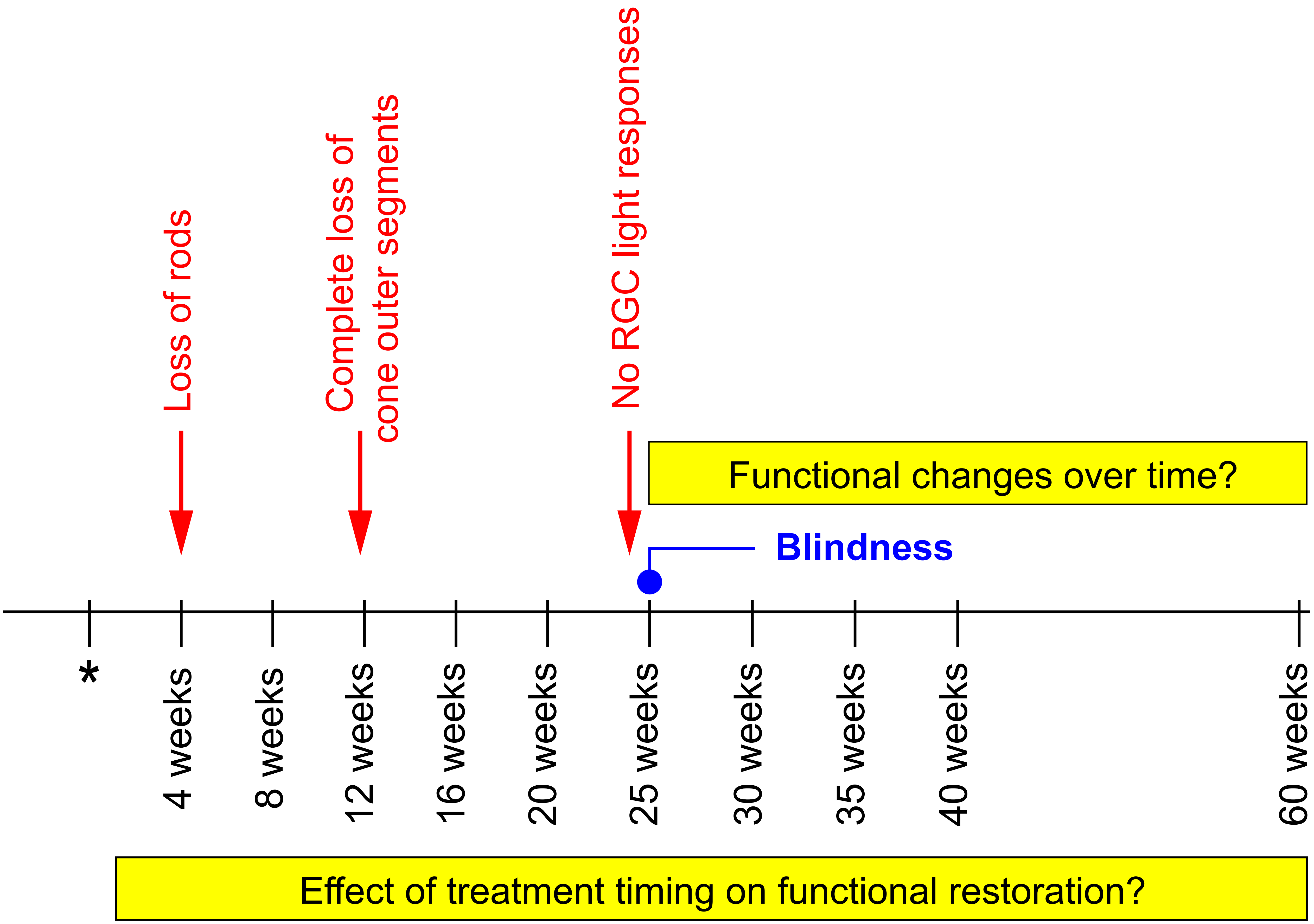

Figure S1. Timeline of retinal degeneration in C3H/HeOu rd1 mice. Loss of rods peaks at 4 weeks of age, followed by complete loss of cone outer segments at week 12, with the peak of cone degeneration happening at 8 weeks of age. Cone cell bodies, however, remain present up to 24 weeks of age – which is the time point when no RGC photoreceptor-derived light responses are observed anymore. Only after this time point can we state that the C3H/HeOu mice are truly blind. Central question of optogenetic vision restoration targeting OBCs is whether there is an effect of time point of treatment onto the functionality of restored vision. Another central question is whether there are functional changes happening in remaining retinal layers over extend periods of time (>25 weeks of age).
