# Supplementary material for: Functional Availability of ON-Bipolar Cells in the Degenerated Retina: Timing and Longevity of an Optogenetic Gene Therapy
Source: Int J Mol Sci. 2021 Oct 26;22(21):11515. doi: 10.3390/ijms222111515 (PMC8584043; doi:10.3390/ijms222111515)
Supplement: Supplementary file 1 [file ijms-22-11515-s001.zip › Figure S2.pdf]

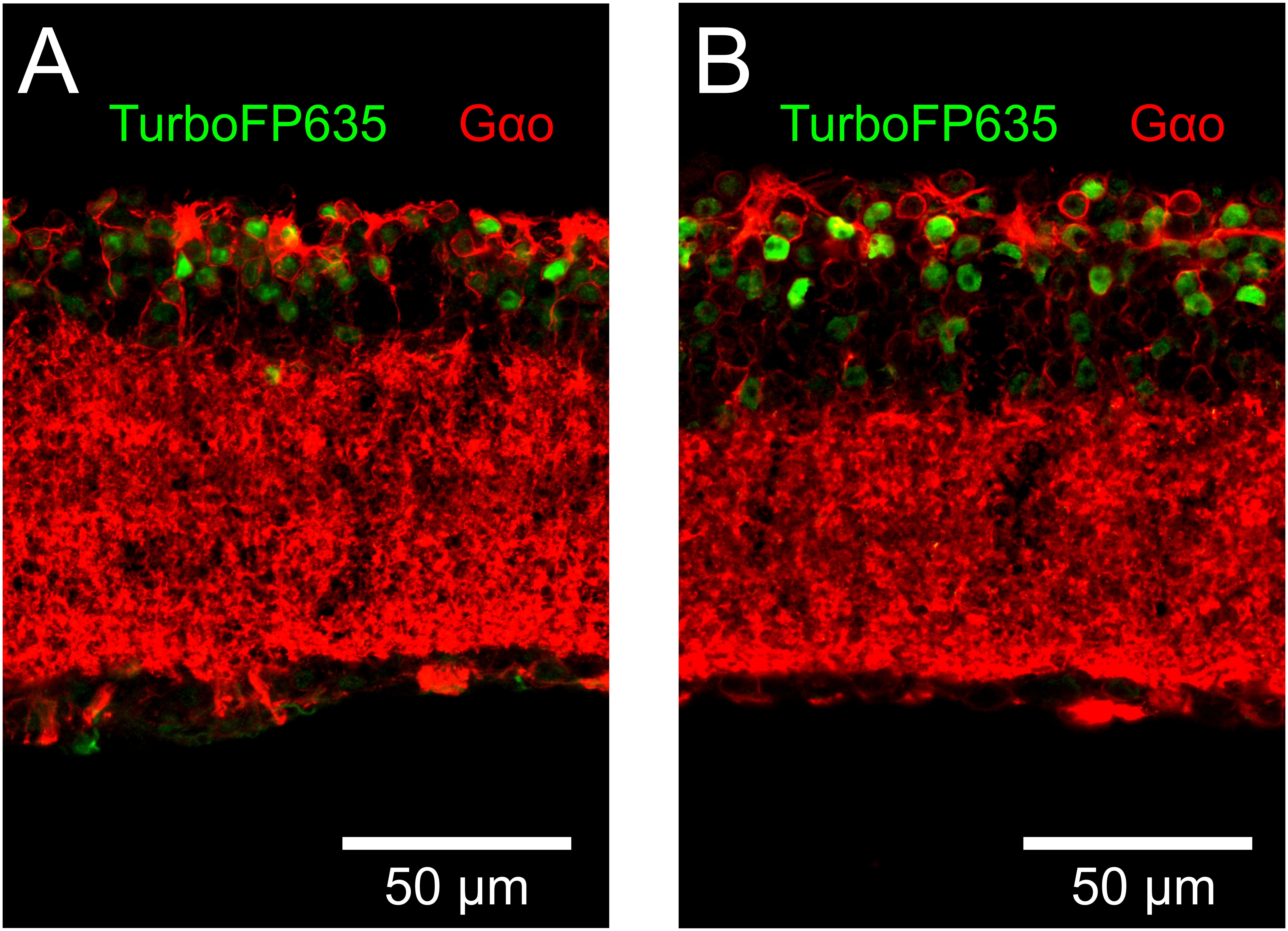

Figure S2. Vertical cryosections of Opto-mGluR6 treated retinal explants were stained against TurboFP635 (green) and Gαo (red). Robust and ON-bipolar cell specific transfection was observed in both younger animals (**A**) evaluated at 32 weeks of age and older animals (**B**) evaluated at 47 weeks of age. Treatment administration was at 3 weeks of age.
